# The Transcriptional Regulatory Network of *Corynebacterium pseudotuberculosis*

**DOI:** 10.3390/microorganisms9020415

**Published:** 2021-02-17

**Authors:** Doglas Parise, Mariana Teixeira Dornelles Parise, Anne Cybelle Pinto Gomide, Flávia Figueira Aburjaile, Rodrigo Bentes Kato, Marisol Salgado-Albarrán, Andreas Tauch, Vasco Ariston de Carvalho Azevedo, Jan Baumbach

**Affiliations:** 1 Chair of Experimental Bioinformatics, TUM School of Life Sciences, Technical University of Munich, 85354 Freising-Weihenstephan, Germany; mparise@wzw.tum.de (M.T.D.P.); marisol.salgado@tum.de (M.S.-A.); jan.baumbach@uni-hamburg.de (J.B.); 2 Institute of Biological Sciences, Universidade Federal de Minas Gerais, Belo Horizonte, Minas Gerais 31270-901, Brazil; acybelle@gmail.com (A.C.P.G.); rbkato@gmail.com (R.B.K.); vasco@icb.ufmg.br (V.A.d.C.A.); 3 Oswaldo Cruz Institute, Fiocruz, Rio de Janeiro, Rio de Janeiro 21040-360, Brazil; faburjaile@gmail.com; 4 Departamento de Ciencias Naturales, Universidad Autónoma Metropolitana Cuajimalpa, Mexico City 05348, Mexico; 5 Center for Biotechnology (CeBiTec), Bielefeld University, 33615 Bielefeld, Germany; tauch@cebitec.uni-bielefeld.de; 6 Computational BioMedicine lab, Institute of Mathematics and Computer Science, University of Southern Denmark, 5230 Odense, Denmark; 7 Chair of Computational Systems Biology, University of Hamburg, 22607 Hamburg, Germany

**Keywords:** *Corynebacterium pseudotuberculosis*, transcriptional regulatory mechanisms, transcription factors, two-component systems, sigma factors, virulence, pathogenicity

## Abstract

*Corynebacterium pseudotuberculosis* is a Gram-positive, facultative intracellular, pathogenic bacterium that infects several different hosts, yielding serious economic losses in livestock farming. It causes several diseases including oedematous skin disease (OSD) in buffaloes, ulcerative lymphangitis (UL) in horses, and caseous lymphadenitis (CLA) in sheep, goats and humans. Despite its economic and medical-veterinary importance, our understanding concerning this organism’s transcriptional regulatory mechanisms is still limited. Here, we review the state of the art knowledge on transcriptional regulatory mechanisms of this pathogenic species, covering regulatory interactions mediated by two-component systems, transcription factors and sigma factors. Key transcriptional regulatory players involved in virulence and pathogenicity of *C. pseudotuberculosis*, such as the PhoPR system and DtxR, are in the focus of this review, as these regulators are promising targets for future vaccine design and drug development. We conclude that more experimental studies are needed to further understand the regulatory repertoire of this important zoonotic pathogen, and that regulators are promising targets for future vaccine design and drug development.

## 1. Introduction

Transcriptional regulation is one of the most important mechanisms of bacterial adaptation to changes in the environment; in particular, pathogenic bacteria use this mechanism to contend the conditions they are exposed to when infecting the host. These conditions are associated with immune barriers imposed by the host to fight the infection; for instance, pH, oxidative and osmotic stresses, temperature and changes in nutrient availability. To quickly adapt, survive and establish the infection, transcriptional regulation controls key biological processes, such as biofilm formation, quorum sensing, temperature sensing and regulation of virulence, are key transcriptional elements of pathogens [1]. For a comprehensive review of bacterial gene regulation mechanisms in general see, for example, [2].

The main players in gene regulation are transcription factors (TFs), which are regulatory proteins that activate or repress the expression of their target genes (TGs) [3,4]. TF-TG relationships can be experimentally investigated using techniques such as RNA-Seq [5], expression microarrays [6], electrophoretic mobility shift assay (EMSA) [7,8], chromatin immunoprecipitation and DNA microarray (ChIP-chip) [9] and chromatin immunoprecipitation and sequencing (ChIP-seq) [10]. RNA-Seq and expression microarrays are used to measure the transcriptional profile of an organism under different conditions; for a comparison of these techniques see [11] and for comprehensive reviews see [12,13,14,15]. EMSA, ChIP-chip and ChIP-seq are used to identify transcription factor binding sites (TFBSs), which are the genomic regions occupied by TFs to regulate gene expression. For comprehensive reviews about ChiP-chip and ChiP-Seq see [16,17,18].

To have a broader view of these TF-TG relationships in systems biology, we model these as directed graphs in which the nodes represent the TFs or the TGs and the arcs, also called directed edges, represent the regulatory interactions, forming what is called transcriptional regulatory network (TRN) [3,4]. Data generated from both experimental and computational techniques are often available from online TRN databases such as RegulonDB [19] for *Escherichia coli* K12, Subtiwiki [20] for *Bacillus subtilis*, Abasy Atlas [21] for *Corynebacterium glutamicum* and CoryneRegNet [22] for the genus *Corynebacterium*. Such computational models have been utilized to understand the transcriptional mechanisms modulating cellular adaptation, survival and pathogenicity of several bacteria species [23,24]. For instance, several studies have demonstrated the role of TFs in the regulation of virulence in pathogenic bacteria such as *Mycobacterium tuberculosis* [25,26,27,28,29], *Pseudomonas aeruginosa* [30] and *Helicobacter pylori* [31]. Despite the experimental and computational advances, there are several understudied pathogenic bacteria that have no efficient treatment, amongst them we can highlight *Corynebacterium pseudotuberculosis*.

*C. pseudotuberculosis* is a pathogenic bacterium that stands out for being an organism of medical and veterinary importance that causes great economic impact on agriculture worldwide. This bacterium is a Gram-positive, facultative intracellular organism that is part of the order Corynebacteriales, which includes species of *Corynebacterium*, *Mycobacterium*, *Nocardia* and others. It is also classified into *equi* and *ovis* biovars, each causing different diseases [32]. The first causes ulcerative granulomatous lesions and mastitis in cattle [33,34], ulcerative lymphangitis (UL) or pigeon fever in horses [35] and oedematous skin disease (OSD) in buffaloes [36]. The second mainly affects small ruminants such as sheep and goats, as well as humans, causing caseous lymphadenitis (CLA) [37]. Furthermore, finding an effective vaccine against these bacteria is still a challenge [38,39]. Despite the importance of *C. pseudotuberculosis*, little is known about the mechanisms that control gene expression. A few studies have evaluated its transcriptional profile across different environmental conditions [40,41,42,43] and a few others have investigated mutant strains showing the importance of specific genes, such as *plD* [44], the *fagABC* operon [45] and *phoP* [46] in *C. pseudotuberculosis* virulence and pathogenicity. Recently, Parise et al. reconstructed in silico TRNs for all *C. pseudotuberculosis* strains with complete genome sequences and made it available in the seventh version of CoryneRegNet [22].

In this review, we collect, summarize and examine the current knowledge of transcriptional regulation of *C. pseudotuberculosis*. First, we present the TRNs and co-expression networks available for this organism. Then, we discuss single-gene studies and “omic” analyses together with the TRNs regarding the presented genes in order to understand the transcriptional regulation of *C. pseudotuberculosis*; these results are presented by distinct regulator types: two-component signal transduction systems (TCSs), TFs and sigma factors. In this way, we aim to summarize and condense our view on *C. pseudotuberculosis* transcriptional regulation by jointly discussing RNA-seq and mutation assays in the light of network biology.

## 2. Gene Co-Expression Networks and Transcriptional Regulatory Networks

To understand how genes interact and influence the expression of other genes, TRNs and gene co-expression networks (GCNs) have been used to analyze bacterial genomes [19,20,47,48,49]. While TRNs present regulatory interactions between regulatory proteins and their targets, GCNs present correlation between the expression of certain genes in the network. Such networks have been used to model how regulatory processes work inside the cell, including amino acid synthesis and virulence mechanisms [50,51,52,53]. Franco et al. [54] and Parise et al. [22] performed GCN analysis and TRN transfer, respectively, in *C. pseudotuberculosis*.

Franco et al. inferred the GCNs of four *C. pseudotuberculosis* strains (258, T1, Cp13 and 1002) using RNA-Seq datasets [40,41,42,43]. The authors applied the following bioinformatic tools: (i) miRsig [55] to infer the GCNs of all genes and differentially expressed genes (DEGs), (ii) miRinfluence [56] to identify the predicted networks’ influential and causal genes and (iii) Online GEne Essentiality (OGEE) database v2 [57] to classify the causal genes as essential, nonessential or conditionally essential [54]. Essential, nonessential and conditionally essential genes demonstrate the consensus of the level of essentiality of a certain gene for bacterial survival, for more details see [57]. The total number of genes and gene-gene interactions of each GCN are presented in Table 1. In the GCN analyses, the following genes were considered causal and categorized as essential: *galU* and *argS* in 258; *pdpB* and *trpC* in T1; and *serC*, *mraY* and *glmS* in Cp13. The above-mentioned genes had not previously been analyzed experimentally in *C. pseudotuberculosis*; however, previous studies in other bacterial species support their relevance for organisms’ growth and survival [58,59,60,61,62,63]. For instance, *galU* increases glycogen and trehalose amounts in *C. glutamicum* [58], and it is also a potential drug target in *M. tuberculosis* [59]. The *argS* gene encodes an arginyl-tRNA synthetase [60], which is induced in the presence of arginine and repressed in the presence of lysine. A study has also proposed that the absence of *argS* is lethal for *Brevibacterium lactofermentum*, because it is not able to synthesize proteins without an arginyl-tRNA synthetase [64]. The *pdpB* gene is homologous of the *lcmF* gene, which is the result of the fusion of the radical B12 enzyme isobutyryl-CoA mutase and its G-protein chaperone [61,65]. In *Francisella*, a deletion mutant of this gene is defective for intracellular bacterial growth [61]. The *trpC* gene, an indole-3-glycerol phosphate synthase [66], is considered essential for growth in both *M. tuberculosis* and *Mycobacterium. bovis* [67], and was identified as a potential drug target in *M. tuberculosis* [59]. The *serC* gene encodes a phosphoserine aminotransferase and is involved in the biosynthesis of L-serine in *C. glutamicum* [68]. In *E. coli* it is induced by cyclic AMP-dependent and together with *aroA* encodes enzymes that lead serine and aromatic amino acids synthesis [69]. The *marY* gene encodes an undecaprenyl-phosphate phospho-*N*-acetylmuramoyl-pentapeptide transferase [70] and is considered essential for cell growth in *E. coli* [63]. It also participates in the building process of the peptidoglycan layer of the cell wall in corynebacteria [71]. The *glmS* gene is a glucosamine-6-phosphate synthase that can enhance *N*-acetylglucosamine synthesis in *C. glutamicum* [62] and was also indicated as a drug target in *M. tuberculosis* in [72,73].

Parise et al. analyzed the conservation of transcriptional regulation in the genus *Corynebacterium* [22]. The authors used experimental TRNs of *M. tuberculosis*, *B. subtilis*, *C. glutamicum* and *E. coli* as models for predicting the TRNs of all complete genomes of this genus, including 91 strains from *C. pseudotuberculosis*. This prediction was performed by checking the conservation of the TFs and the TGs with BLAST software [74] and the conservation of the TFBSs with HMMER package [75]. These predictions are publicly available in CoryneRegNet 7, which holds 24,069 regulatory interactions, 2990 TFs and 17,298 TFBSs for this species. Such networks will be discussed and presented alongside literature data regarding *C. pseudotuberculosis* regulations in the following sections.

## 3. Regulators of Gene Expression

### 3.1. Two-Component Systems

Two-component signal transduction systems (TCS) detect and mediate the response to external stimuli by means of a series of biochemical signals that result in gene expression changes [46,76]. These processes regulate many processes such as drug resistance, adhesion, sporulation, pilus formation, cell division, nutrient acquisition, nitrogen fixation and virulence [77,78,79,80].

One of the most well-known TCS is the PhoPR system, in which a transmembrane sensory histidine kinase protein (PhoR) phosphorylates the receiver domain of the response regulator protein (PhoP). The phosphorylation of PhoP results in the activation of the effector domains, which causes a transcriptional response. Some studies have used *phoP* mutant strains as vaccinal strategies, not only in *C. pseudotuberculosis*, but also in *M. tuberculosis* [46,81]. In *C. pseudotuberculosis*, these mutant strains presented reduced virulence in mice and induced a host cellular immune response [46]. Additionally, the absence of the *phoP* gene resulted in higher levels of IgG antibodies in contrast with the control group.

**Figure 1:**
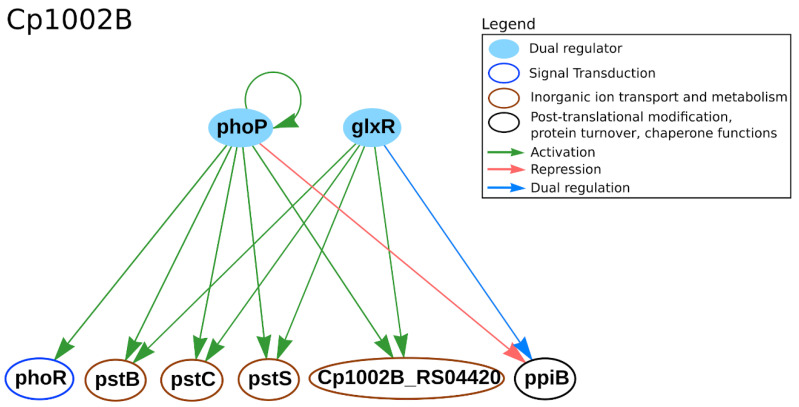
PhoPR transcriptional regulatory network (TRN) retrieved from CoryneRegNet 7 [22] for *C. pseudotuberculosis* 1002B. In the network, nodes represent the genes and arrows represent the regulatory interactions. The functional classification was performed using the database Eggnog (evolutionary genealogy of genes: non-supervised orthologous groups) [90].

In CoryneRegNet 7 [22], the predicted TRN of the *phoP* and *phoR* genes in *C. pseudotuberculosis* 1002B strain is shown in Figure 1. The TFs *phoP* and *glxR* jointly activate the phosphate ATP-binding cassette (ABC) transporters *pstB*, *pstC*, *pstS* and Cp1002B_RS04420 (frameshift *pstA*). The *ppiB* gene is repressed by *phoP* and is dually regulated by *glxR*, while *phoR* is activated by *phoP*. Due to its crucial role in bacterial survival, development and adaptation to environmental changes, the PhoPR system is an interesting drug target for future studies [46,82]. In *C. glutamicum,* the *pstSCAB* operon, an ABC transport system for the uptake of phosphate, is induced during phosphate starvation [83]. The *ppiB* gene is a peptidyl-prolyl cis/trans isomerase (PPIase); it is important for growth in both low temperatures and starvation conditions in *Legionella pneumophila* [84] and *B. subtilis* [85]. PPIases were marked as alternative drug targets [86]. Both the *pstSCAB* operon and the PPIases were already indicated as bacterial virulence factors in *L. pneumophila* [86] and *Salmonella* [87,88,89].

The *hrrA* gene is part of the HrrSA TCS and it was differentially expressed in both *C. pseudotuberculosis* T1 and CP13 under iron starvation [43]. This system both activates the expression of a heme oxygenase (*hmuO* gene) and represses genes acting in heme homeostasis in *C. glutamicum* and *Corynebacterium diphtheriae* [91,92], and is considered the global regulator of heme in *C. glutamicum* [93]. In this system the HrrS is the sensor kinase and the HrrA is the response regulator. Furthermore, Franco et al. [54] identified three TCS genes, namely, *tcsS4*, *mprA_2* and *tcsR3,* as influential genes. However, they remain to be studied; there is no regulatory information in CoryneRegNet 7 for the HrrSA TCS and the three genes found by Franco et al.

### 3.2. Transcription Factors

TFs can modulate gene expression through activating or repressing transcription by different mechanisms. In all mechanisms, activation enhances the interaction between the RNA polymerase and the promoter, and repression prevents their interaction [94]. In bacteria, the environmental signals are the elements responsible to modulate TFs activity influencing transcription initiation [95]. In this section we describe the TFs that perform key functions in *C. pseudotuberculosis* in the context of the biological mechanism they are involved in.

### 3.3. Metalloregulation: Iron Uptake

Metal ions are essential for bacterial metabolism; in particular, iron, manganese and zinc are used as cofactors [96]. Iron is an important protein cofactor required for growth and development in virtually all living organisms; the acquisition of host iron is a well-characterized mechanism of infections used by bacterial pathogens to successfully establish the infection in host cells. Besides its importance, an excess of iron can be toxic to the cell; thus, in order to keep iron homeostasis, bacteria developed a tightly-regulated system [43,97].

**Figure 2:**
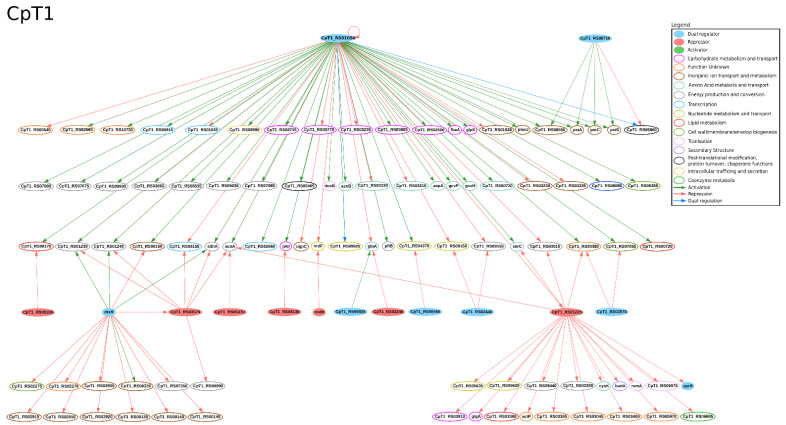
Regulatory interactions taken from CoryneRegNet 7 [22] for *C*. *pseudotuberculosis* T1 under iron limitation in [43]. In the network, nodes represent the genes and arrows represent the regulatory interactions. The functional classification was performed using the database Eggnog [90].

**Figure 3:**
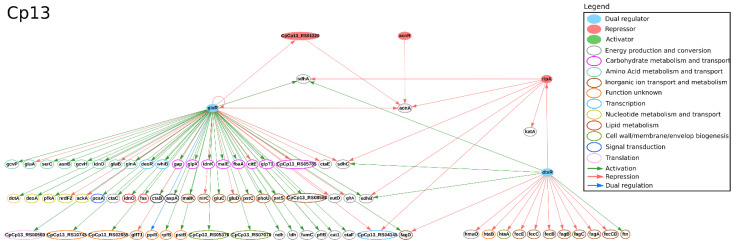
Regulatory interactions taken from CoryneRegNet 7 [22] for *C*. *pseudotuberculosis* Cp13 under iron limitation in [43]. In the network, nodes represent the genes and arrows represent the regulatory interactions. The functional classification was performed using the database Eggnog [90].

A recent study used RNA-Seq to analyze *C. pseudotuberculosis*-infected spleens of dairy goats and found many iron-related genes differentially expressed in order to reduce iron availability. The authors hypothesized that *C. pseudotuberculosis* evolved an iron acquisition mechanism to manage this reduction [98]. The expression of DtxR, the master regulator of iron, is downregulated under iron limitation, directly and indirectly influencing the expression of several genes in *C. pseudotuberculosis* [43]. In CoryneRegNet, this TF is predicted to regulate sixteen genes in both Cp13 and CpT1 [22]; such regulatory interactions are presented in Figure 2 and Figure 3, respectively. Some of DtxR’s target genes are the *fagABC* operon and the *fagD* gene, which are components of the iron acquisition system and important virulence factors of this organism [40,45]. Studies in *C. diphtheriae* and *C. pseudotuberculosis* indicate that DtxR regulates the *ciuA* gene [99,100]; however, a recent study [43] in *C. pseudotuberculosis* T1 and Cp13 found no difference in the expression of these genes under iron restricted conditions. Likewise, DtxR is not predicted to regulate the gene *ciuA* in CoryneRegNet [22]. The authors of the iron-limitation study [43] used the Ion Proton platform to analyze the transcriptome of the wild-type *C. pseudotuberculosis* strain T1 and its mutant strain, Cp13, which has a disrupted *ciuA* gene. The *ciuA* gene encodes a protein highly similar to siderophore ABC-type transport systems and has been previously associated with virulence [101]. The lack of this gene resulted in reduced growth [43] and intracellular viability [102] of the mutated strain. In the same study, 77 and 59 DEGs were identified in T1 and Cp13, respectively. Besides that, the authors observed an up-regulation of hemin acquisition systems and down-regulation of iron intracellular utilization in both strains. The expression of hemin uptake systems in Cp13 may indicate the adaptive response of the transcription machinery to iron acquisition from other sources. Hemin uptake genes were found in genomic islands together with many known virulence factors, corroborating previous studies pointing to the association of iron uptake and virulence in *C. pseudotuberculosis* [43,45,102].

In the same study, iron restriction increased the expression of genes associated with putative hemin acquisition systems and decreased the expression of genes associated with energy metabolism in both strains. Down-regulated genes involved in the oxidative phosphorylation process and tricarboxylic acid cycle (TCA) cycle were only found in the T1 strain. Amongst them, *sdhA*, *sdhB* and *sdhC* genes encode succinate dehydrogenase iron-sulfur proteins, which compose the respiratory complex II. In this complex, the succinate reduction links the oxidative phosphorylation process with the TCA. These genes are predicted to be jointly regulated by GlxR, DtxR and RipA. Regarding the TFs, Ibraim and collaborators [43] found the following up-regulated genes: *ripA* in both strains; *glpR*, *cspA*, *whiB* and *sufR* in Cp13; and *glxR* in T1. In CoryneRegNet, only *ripA* and *glxR* regulate other genes in these strains [22]. *RipA* encodes a protein that belongs to the AraC family regulators that repress the expression of genes encoding iron-containing proteins. This TF is predicted to be regulated by DtxR and to regulate six genes in both T1 and Cp13 in [22]; the *sdhA*, *sdhB* and *sdhC* genes were identified as differentially expressed in the iron limitation assay [43]. GlxR is a global regulator involved in the regulation of metabolic processes [91,92]. It is predicted to regulate 79 genes in both T1 and Cp13 [22], including two TFs: *ramB* homologue (CpCp13_RS01220 and CpT1_RS01225) and *whiB* homologue (CpCp13_RS01035, CpCp13_RS02650 and CpT1_RS02660) in both strains. The predicted regulatory networks of the DEGs found under iron limitation in T1 and Cp13 are presented in Figure 2 and Figure 3, respectively [22]. The regulators RamB, TetR family protein (CpT1_RS08165), AcnR homologue (CpT1_RS05370), NrdR, AmtR homologue (CpT1_RS03240), Ram*A* homologue (CpT1_RS08465), RbsR homologue (CpT1_RS02440), MtrA homologue (CpT1_RS02575) and PyrR are present in the regulatory network and were not identified in the experimental assays, which indicated that they may be involved in other cellular functions other than metalloregulation and need to be further investigated.

### 3.4. Response to Osmotic, Thermal and Acid Stress

Bacteria experience stress conditions not only when migrating from the environment to the host, but also when invading and colonizing the host’s bloodstream, gastrointestinal and respiratory tracts, mucous membranes and immune system [103,104,105]. In order to survive these conditions, the organism must assemble a quick protective response at the transcriptional level [105,106]. During environmental changes, a reduction, or even a lack of growth, is considered normal in bacteria [40,107]. Three studies in *C. pseudotuberculosis* point out a reduction of replication of ~23%, ~27% and ~34% in strain 1002 and 16%, 20% and 36% in strain 258 under osmotic, thermal and acid stresses, respectively [40,41,42]. The first study performed a differential expression analysis under these conditions in *C. pseudotuberculosis* 1002 and identified DEGs involved in oxidoreduction, adhesion and cell division processes [40]. The other two allow us to further understand the transcriptional response induced during these stresses in *C. pseudotuberculosis* 258 [41,42]. In all three studies, the authors performed transcriptome analyses using the SOLiD 3 Plus platform [40,41,42]. A notable TF identified in these two strains is TetR2, a DEG found across the three stress conditions. This TF belongs to the TetR family that in general regulates the expression of genes involved in drug resistance, biosynthesis of antibiotics, pathogenicity, virulence, quorum sensing and catabolic pathways [40,41,42,108]. So far, there is no experimentally verified or predicted regulation for this TF in *C. pseudotuberculosis* 1002B and 258.

Under acid stress the following DEGs were highly expressed: *msrB* in strain 1002B, *msrA in* strain 258 and both *dps* and *lysR1* in these two strains [40,42]. In CoryneRegNet, the TF LysR1 is predicted to repress itself and activates the expression of the peroxiredoxin gene *ahpC* in both strains. Gomide et. al. 2018 suggests that this TF plays a modulatory role in *C. pseudotuberculosis* 258; however, no experimentally verified LysR1 regulation is known in these strains [42]. In *P. aeruginosa,* a LysR-family TF regulates the expression of genes related to virulence and stress response modulators [109]. The *msrB* and *msrA* gene products act together resulting in the catalytic activity of the oxidation-reduction of methionine sulfoxide. The *msrA* gene plays a more relevant role in virulence than *msrB* does in bacteria [42,110]; it corroborates the fact that *C. pseudotuberculosis* strains from the *equi* biovar, such as 258, are more virulent than the ones from the *ovis* biovar, such as 1002B. The *dps* gene protects the bacteria under acid, oxidative and heat stresses, as well as in iron and copper toxicity. The inactivation of this gene in *E. coli* leads to a reduction in the survival rate of the bacteria in an acid environment [42,111,112]. The *dps*, *msrA* and *msrB* genes have no regulatory interactions predicted in CoryneRegNet [22] and are interesting candidates for future experimental assays.

**Figure 4:**
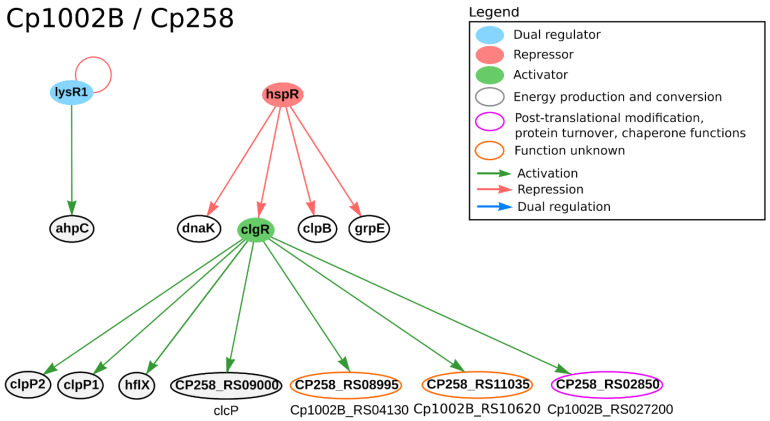
Regulatory interactions from CoryneRegNet 7 for the DEGs of *C. pseudotuberculosis* 258 (Cp1002B) and 1002B (Cp258) under osmotic, acid and heat stress conditions [41,42]. In the network, nodes represent the genes and arrows represent the regulatory interactions. The functional classification was performed using the database Eggnog [90].

Under thermal shock stress, *hspR*, *dnaK* and *grpE* genes were differentially expressed in both organisms. The *hspR* gene, which encodes a heat shock TF, is known for regulating genes involved in virulence and pathogenicity [40,113]. Additionally, it regulates heat shock operons, which encode genes that maintain the structure of proteins in several cell stresses [114,115]. Likewise, in 1002B and 258 it is predicted to regulate four genes: *dnaK*, *grpE*, *clpB* and *clgR*. The *dnaK* gene improves the immune response in the host and seems to regulate genes encoding virulence factors and bacterial adhesion [40,115]. The *grpE* gene is also a chaperone involved in bacterial virulence and belongs to the same operon as *hspR* and *dnaK* [41]. The *clpB* gene was differentially expressed under both thermal and osmotic stresses in the strain 258. It encodes an ATP-dependent chaperone that is involved in virulence and participates in the stress response system [116,117]. The *clgR* gene regulates the expression of genes acting in DNA repair and proteolysis in *C. glutamicum* [118]. In CoryneRegNet, it is predicted to regulate seven genes in *C. pseudotuberculosis* 1002B and 258, as presented in Figure 4.

Under osmotic stress the following DEGs were highly expressed: *glmU* and *uppP* in strain 258 and *norM* in strains 1002B and 258 [40,42]. The *glmU* gene encodes an enzyme that catalyzes the substrate in the synthesis of bacterial peptidoglycans and lipopolysaccharides of the cell wall [119]; it is predicted to be regulated by the *glk* homologue (CP258_RS07175) in *C. pseudotuberculosis* 258 in CoryneRegNet [22]. A study in *Mycobacterium smegmatis* showed that *glmU* is both a drug target and crucial for bacterial replication [120]. The *uppP* gene encodes an enzyme that is involved in the biosynthesis of both membrane proteins and bacterial cell wall components, a process that is essential for bacterial integrity [121,122]. This also makes the *uppP* gene crucial to bacterial growth and bacterial pathogenicity, making it an interesting drug and vaccine target, as well [42,120]. The *norM* gene is a multidrug efflux pump that belongs to an ABC transporter family, conferring organisms an effective antibiotic resistance [42,123,124]. In *C. pseudotuberculosis* 258, the *srtA* gene was differentially expressed under both osmotic and thermal stresses. The *srtA* gene encodes a sortase, which is a housekeeping gene involved in the pathogenesis and virulence of Gram-positive bacteria. It contributes to the covalent binding of the peptidoglycan layer and cell surface proteins [41,42,125,126]. There are no regulatory interactions for *uppP*, *norM* and *srtA* genes in CoryneRegNet [22] for these strains, such genes are promising candidates for experimental assays. Figure 4 presents the regulatory interactions from CoryneRegNet of the genes found as DEGs in *C. pseudotuberculosis* 258 and 1002B (Figure 4) strains under osmotic, acid and heat stress conditions [40,41,42].

### 3.5. Sigma Factors

In prokaryotes, one of the most important stages of the gene expression regulation is the initiation of the transcription. During this stage, sigma factors are both required to assemble the RNA polymerase holoenzyme and to recognize the promoters [127,128]. Similar to TFs, sigma factors are key players in transcriptional regulation when adapting to stress conditions, such as osmotic, thermal, acid and nutrient starvation stresses [129,130]. These molecules are also known to be involved in the regulation of virulence genes [131,132]. Bacterial sigma factors include SigA, SigB, which are essential and nonessential, respectively, and the alternative sigma factors SigC, SigD, SigE, SigH, SigK and SigM. These alternative sigma factors may belong to the extracytoplasmic factors group [133,134], which is responsible for the regulation of genes involved in the transport, cellular wall adaptation or secretion within the periplasm (Gram-negatives) or extracellular environment [131].

In *C. pseudotuberculosis* 1002 there are eight genes encoding sigma factors [135], whereas in *C. glutamicum*, a nonpathogenic bacteria, there are seven sigma factors [134]. In particular, the *sigK* gene is present only in C. *pseudotuberculosis*, which suggests that it may have a role in the virulence mechanisms of *C. pseudotuberculosis*. The study of Pinto et al. [40] observed the expression changes of some sigma factors in *C. pseudotuberculosis* 1002 in the beginning of the exponential phase under heat, osmotic and acid stresses, simulating host-infection conditions. The sigma factors analyzed in the aforementioned study are shown in Table 2.

Considering the authors’ fold-change threshold of 2×, the following sigma factors were differentially expressed: *sigA* under osmotic stress; *sigB*, *sigE* and *sigH* under acid stress; and *sigM* in all conditions. In *C. pseudotuberculosis* 1002, the *sigA* and *sigH* genes, which encode RpoD and RpoE sigma factors, respectively, were significantly differentially expressed in all three conditions. The *sigA* gene, also known as sigma 70, promotes the binding of the RNA polymerase to specific sites activating the transcription of most essential genes related to the exponential growth in *E. coli* [136]. In *C. pseudotuberculosis* 1002, this protein conserves the four domains belonging to sigma 70. The *sigB* gene was considered to be induced in the same study and is known to regulate genes involved in the stress response of many Gram-positive bacteria. Both *sigA* and *sigB* were previously associated with virulence in other bacteria; *S*igA can be specifically required for the expression of virulence genes in *M. tuberculosis* [131,137]. SigB controls the expression of many genes involved in the virulence of pathogens, such as biofilm formation, cellular differentiation, pathogenesis, stress resistance and sporulation in several bacteria [131,138]. Pacheco et al. assessed the role of SigE in *C. pseudotuberculosis* using a *sigE*-mutant strain under different stress conditions, including acid stress. The authors observed a higher in vitro susceptibility of this bacteria in the host-simulated conditions, inferring the importance of SigE in the bacterial maintenance within the unfavorable environment [139].

**Figure 5:**
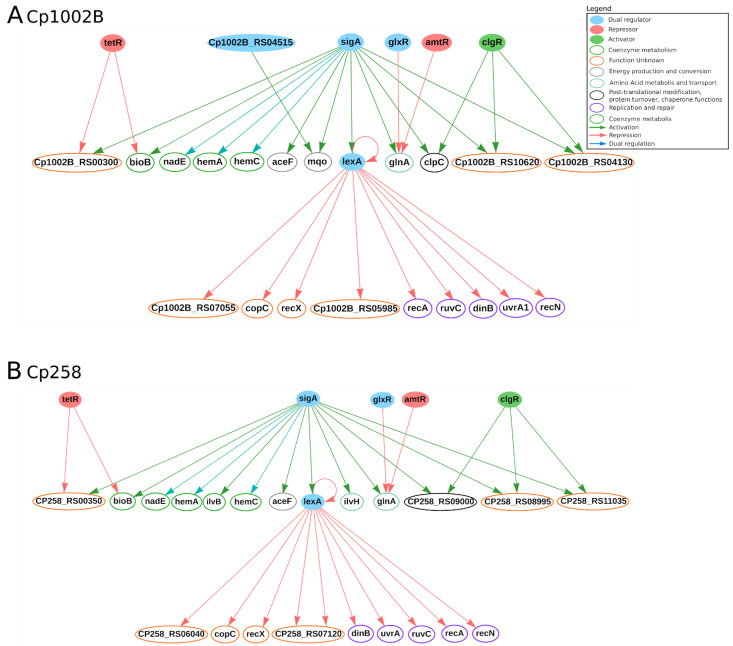
Regulatory interactions of SigA in *C. pseudotuberculosis* 1002B (**A**) and *C. pseudotuberculosis* 258 (**B**) from CoryneRegNet 7. In the network, nodes represent the genes and arrows represent the regulatory interactions. The functional classification was performed using the database Eggnog [90].

Interestingly, no significant expression changes in these molecules were observed in both 258, under the same three stress conditions applied to *C. pseudotuberculosis*, and Cp13 and T1, under iron starvation [41,42,43]. For all sigma factor coding genes, it is necessary to unravel the regulon, as well as unveil all the interaction network coding genes of the sigma factors for a better understanding of the infection process and response modulation in the cell. The predicted regulatory interactions in CoryneRegNet of the sigma factors mentioned in this section are presented in Figure 5A,B for strains 1002B and 258, respectively. Finally, the function of experimentally studied sigma factors in *C. glutamicum* and *M. tuberculosis* help to provide clues on their regulatory roles in *C. pseudotuberculosis* [133,136].

## 4. Conclusions

In this review, we presented the current knowledge of the landscape of *C. pseudotuberculosis* transcriptional regulation. The behavior of this organism under osmotic, acid, iron-starvation and thermal stress was studied exemplarily as well. We conclude that we have just begun to understand the importance of some key transcription factors, such as PhoP, DtxR, Rip*A* and GlxR, as well as of some of the sigma factors. Apart from that, very little is known about the regulatory mechanisms of this organism. New RNA-seq analyses under several conditions and preferably also time-series data combined with other layers of regulatory data are still needed to unravel the pathogenicity, survival and adaptation of *C. pseudotuberculosis* in its diverse range of hosts. Such studies might contribute not only to correctly diagnosing and treating the diseases caused by this organism, but also to identifying better drugs and vaccine candidates based on regulatory pathomechanisms.

## Figures and Tables

**Table 1:** Gene co-expression network (GCN) data from Franco et al. [54]. DEGs -differentially expressed genes.

Strain	Technology	GCN from All Genes		GCN from DEGs		Reference
		Genes	Interactions	Genes	Interactions	
Cp13	Ion Proton	2113	86,367	63	46	[43]
T1	Ion Proton	2093	107,202	93	98	[43]
1002	SOLiD	2091	6682	168	155	[40]
258	SOLiD	2064	9376	139	165	[41,42]

**Table 2:** Fold-change values of the genes encoding sigma factors in osmotic (2 M), heat (50 °C) and acid stresses (pH), from Pinto et al. [40]. °C-degree Celsius. ECF-Extracytoplasmic function.

Sigma Factor	Product	Osmotic Stress		Thermic Stress		Acid Stress	
		Fold-Change	DEG	Fold-Change	DEG	Fold-Change	DEG
*sigA*	RNA polymerase sigma factor SigA (essential housekeeping sigma factor)	2.1889	Yes	1.4903	No	0.9232	No
*sigB*	RNA polymerase sigma factor SigB (non-essential SigA-like)	0.6348	No	0.9044	No	2.9154	Yes
*sigC*	RNA polymerase sigma factor SigC (ECF family)	0.4675	No	0.8031	No	1.7238	No
*sigD*	RNA polymerase sigma factor SigD (ECF family)	1.5437	No	1.2891	No	0.8654	No
*sigE*	RNA polymerase sigma factor SigE (ECF family)	0.5483	No	0.9356	No	2.5244	Yes
*sigH*	RNA polymerase sigma factor SigH (ECF family)	1.8401	No	1.7864	No	3.5832	Yes
*sigK*	RNA polymerase sigma factor SigK (ECF family)	1.5887	No	1.7415	No	1.6199	No
*sigM*	RNA polymerase sigma factor SigM (ECF family)	4.7414	Yes	3.5593	Yes	4.4934	Yes

## Data Availability

Data sharing not applicable.
